# Histone modification cross-talk and protein complex diversification confer plasticity to Polycomb repression

**DOI:** 10.1101/gad.353148.125

**Published:** 2026-01-01

**Authors:** Jacques Bonnet, Eva Triantopoulou, Jasmin Birnhäupl, Chenggang Lu, Margaret T. Fuller, Jürg Müller

**Affiliations:** 1 Laboratory of Chromatin Biology, Max-Planck Institute of Biochemistry, 82152 Martinsried, Germany;; 2 Department of Developmental Biology, School of Medicine, Stanford University, Stanford, California 94305, USA

**Keywords:** PRC1, PCGF proteins, L(3)73Ah, Psc, PRC2, PR-DUB

## Abstract

Polycomb chromatin domains are chromosomal regions decorated with histone H2A monoubiquitination at lysine 119 (H2Aub1) and histone H3 trimethylation at lysine 27 (H3K27me3). These domains are dynamically shaped through the actions of different Polycomb group protein complexes to control gene expression during development. To assess how different Polycomb group subcomplexes contribute to these histone modification profiles in *Drosophila* embryos, we used mutants that abrogate their function. Canonical Polycomb repressive complex (PRC) 1 deposits low levels of H2Aub1 solely at Polycomb target genes, whereas variant PRC1 generates the bulk of H2Aub1 genome-wide. In late-stage embryos, PR-DUB-mediated deubiquitination effectuates a uniform low-level H2Aub1 profile across the genome. The combined activities of PRC2.1 and PRC2.2 drive the formation and maintenance of most H3K27me3 domains, but PRC2.1 is the limiting enzyme for creating such domains at HOX genes. Surprisingly, reduction in the H3K27me3 level and repression defects caused by removing PRC2.1 were largely rescued in animals also lacking PR-DUB, which showed extensive H2Aub1 accumulation at Polycomb targets that promoted compensatory H3K27me3 deposition by PRC2.2. Diversification of Polycomb protein complexes combined with feedback loop mechanisms involving histone modification cross-talk equips the system with the plasticity, adaptability, and buffering capacity needed to safeguard cell fate decisions during development.

## Abstract (precis)

In this study, Bonnet et al. delineate the roles of different Polycomb group subcomplexes in regulating histone modification during embryonic development in *Drosophila*. They show that canonical and variant Polycomb repressive complexes function concertedly to modulate H2Aub1 and H3K27me3 deposition, both genome-wide and at specific target loci, allowing for the formation of functional Polycomb-repressed chromatin.

The evolutionarily conserved Polycomb group (PcG) proteins are subunits of distinct protein complexes that function to maintain cell fate decisions by repressing transcription of genes that regulate development in cells where these genes need to remain inactive. Following genetic studies that identified the PcG genes based on their required role in the long-term repression of HOX genes in *Drosophila*, purification and biochemical characterization of the encoded proteins resulted in isolation of four principal types of complexes: canonical Polycomb repressive complex 1 (cPRC1), Polycomb repressive complex 2 (PRC2), Pho repressive complex (PhoRC), and the Polycomb repressive deubiquitinase (PR-DUB) complex (Shao et al. 1999; Cao et al. 2002; Czermin et al. 2002; Kuzmichev et al. 2002; Müller et al. 2002; Klymenko et al. 2006; Scheuermann et al. 2010). cPRC1 compacts chromatin and possesses E3 ligase activity for monoubiquitination of histone H2A at lysine 118 in *Drosophila* and the corresponding lysine 119 in vertebrates (H2Aub1), PRC2 is the histone methyltransferase for lysine 27 in histone H3 (H3K27me3), and PR-DUB deubiquitinates H2Aub1 (Francis et al. 2001, 2004; Cao et al. 2002; Czermin et al. 2002; Kuzmichev et al. 2002; Müller et al. 2002; Wang et al. 2004; Scheuermann et al. 2010; Grau et al. 2011). PhoRC contains no enzymatic activity but is the only PcG complex with sequence-specific DNA-binding activity and anchors the other complexes at Polycomb target genes (Klymenko et al. 2006; Frey et al. 2016).

Subsequent studies revealed that in addition to the originally characterized canonical PRC1 (cPRC1), both *Drosophila* and mammals contain different forms of PRC1, which were named variant PRC1 (vPRC1) to distinguish them from cPRC1 (Lagarou et al. 2008; Gao et al. 2012; Tavares et al. 2012; for review, see Tamburri et al. 2024). cPRC1and vPRC1 share one common subunit: the protein Sce in *Drosophila* and the corresponding protein Ring1b or its paralog, Ring1a, in mice (Fig. 1A). Sce and Ring1a/b are E3 ligases that must associate with a second Ring finger protein of the PCGF family to monoubiquitinate H2A on nucleosomes (Buchwald et al. 2006; McGinty et al. 2014; Taherbhoy et al. 2015). *Drosophila* has three PCGF proteins. Psc and Su(z)2 are duplicated, partially redundant paralogs that interact with Sce in a mutually exclusive manner and, together with the subunits Polycomb (Pc), Polyhomeotic (Ph), and Sex combs on midleg (Scm), form cPRC1 (Fig. 1A; Shao et al. 1999; King et al. 2005; Lo et al. 2009). The third PCGF protein, Lethal(3)73Ah [L(3)73Ah], is thought to partner with Sce to form the core of two distinct vPRC1 assemblies in *Drosophila* that resemble PRC1.1 and PRC1.3/5 in mammals (Kang et al. 2022). Specifically, affinity purification of L(3)73Ah from formaldehyde-cross-linked nuclei from *Drosophila* embryos identified additional complex components orthologous to subunits in human PRC1.1 and PRC1.3/5 (Fig. 1A; Kang et al. 2022). Rybp is a shared component of all vPRC1 variants, integrating into the complex via interaction with the C terminus of Sce, whereas in cPRC1, the Pc subunit occupies this position by binding to the Sce C-terminal region instead (Wang et al. 2010; Gao et al. 2012; Tavares et al. 2012).

**Figure 1.**
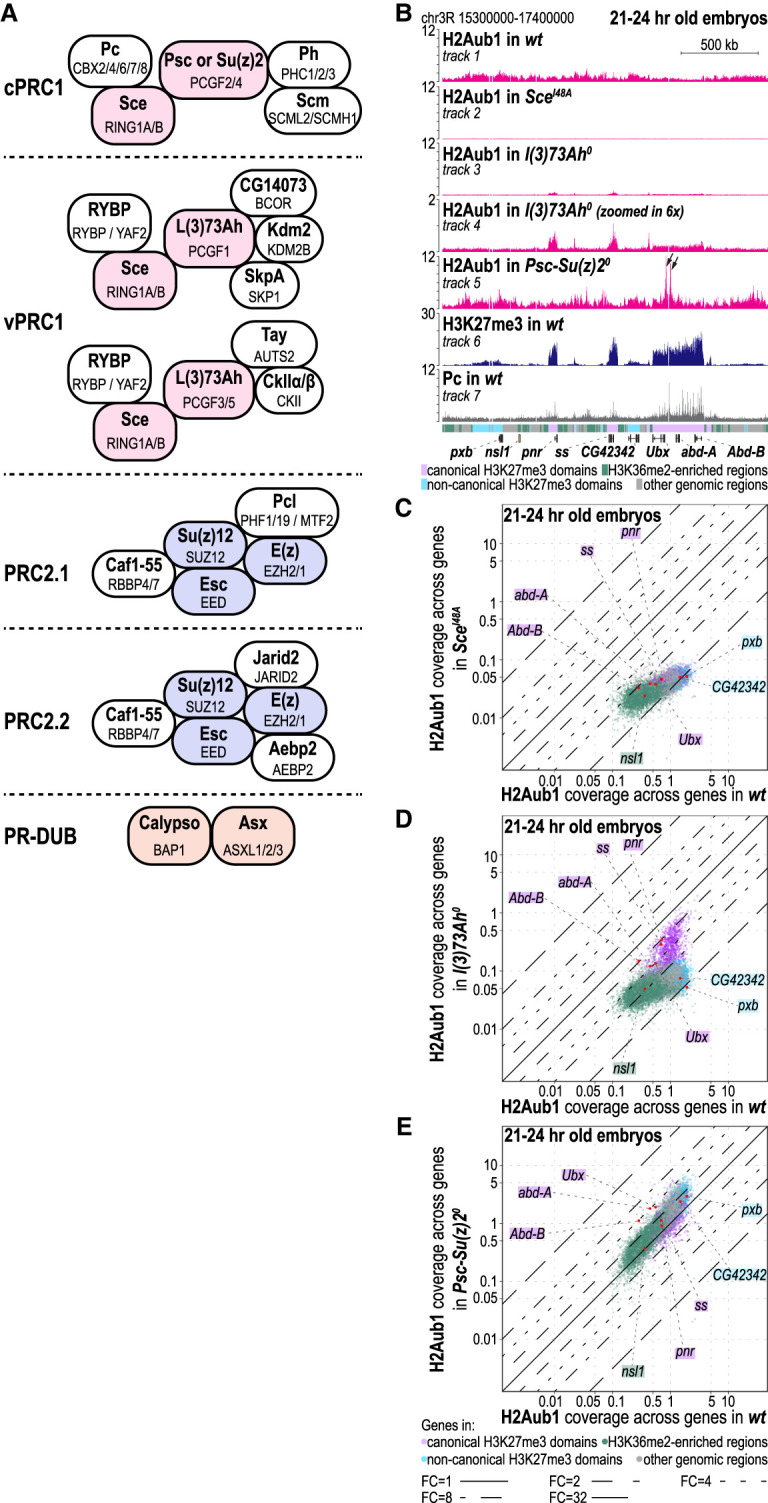
vPRC1 generates the bulk of genome-wide H2Aub1. (*A*) Schematic representation of *Drosophila* and human PRC1 and PRC2 subcomplexes and PR-DUB. For each subunit, the names of the *Drosophila* protein (bold) and the human ortholog (not bold) are indicated. Core complex subunits required for enzymatic activity are shown in color. (*B*) H2Aub1 (tracks 1–5), H3K27me3 (track 6), and Pc (track 7) ChIP-seq profiles in 21–24 h old embryos of the indicated genotypes. The colored bar *below* track 7 shows genomic intervals defined as canonical H3K27me3 domains (pink), noncanonical H3K27me3 domains (light blue), or regions enriched for H3K36me2 (green); all other regions are marked in gray. See Supplemental Figure S2B and the Materials and Methods for details about how these genomic intervals were defined. Locations of the *Bithorax* complex with *Ubx, abd-A*, and *Abd-B*, as well as other genes in this chromosomal interval, are indicated. Arrows in track 5 marks *Ubx* regions with de novo enrichment of H2Aub1 (see the text). (*C*) Scatter plots showing H2Aub1 read coverage across gene bodies in 21–24 h old wild-type (wt) and *Sce*^*I48A*^ mutant embryos. Each dot corresponds to a gene, and the color indicates the chromatin type in which the gene is located. Genes shown in *B* are marked as red dots, and the chromatin color code is indicated in the box around the gene name. Onefold, twofold, fourfold, eightfold, and 32-fold changes are indicated by the FC = 1, FC = 2, FC = 4, FC = 8, and FC = 32 lines, respectively (shown *below E*). (*D*) Scatter plot as in *C* but comparing wild-type and *l(3)73Ah*^*0*^ mutant embryos. (*E*) Scatter plot as in *C*) but comparing wild-type and *Psc-Su(z)2*^*0*^ mutant embryos.

The originally identified PRC2 core complex is present in two distinct assemblies that are called PRC2.1 and PRC2.2, each defined by its association with specific accessory factors (for reviews, see Laugesen et al. 2019; Yu et al. 2019). In *Drosophila*, the PRC2 core is formed by the subunits E(z), Su(z)12, Esc, and Caf1-55 (Fig. 1A; Czermin et al. 2002; Müller et al. 2002). *Drosophila* PRC2.1 contains Polycomblike (Pcl) as an accessory subunit bound to the PRC2 core, whereas PRC2.2 incorporates the accessory factors Jarid2 and Aebp2 (also known as jing) into the core complex (Fig. 1A; Nekrasov et al. 2007; Herz et al. 2012). These PRC2 accessory factors act through distinct molecular pathways: Pcl binds DNA in a sequence-independent manner to prolong the residence time of PRC2.1 on chromatin and thereby facilitates H3K27 trimethylation (Choi et al. 2017), whereas Jarid2 and Aebp2 recognize H2Aub1 and thereby promote H3K27 trimethylation on H2Aub1 nucleosomes (Kalb et al. 2014; Cooper et al. 2016; Kasinath et al. 2021).

In mammals, studies on the different forms of PRC1 or PRC2 have focused primarily on their role in shaping the H2Aub1 and H3K27me3 genomic profiles in human induced pluripotent stem cells (Youmans et al. 2021), in naive or primed mouse embryonic stem cells (Zhao et al. 2017; Fursova et al. 2019; Healy et al. 2019; Højfeldt et al. 2019), or in mouse embryonic stem cells subjected to differentiation into neural progenitor cells (NPCs) (Petracovici and Bonasio 2021). Collectively, these studies demonstrated that the contributions of the various PRC1 and PRC2 complexes to these profiles vary depending on the cellular model used, suggesting that utilization of these complexes may be dynamically regulated during development (for review, see Kim and Kingston 2022). Indeed, work in developing mice and *Drosophila* revealed that Polycomb complex binding and H3K27me3 and H2Aub1 enrichment at target genes often undergo extensive changes during development. A prime example is the H2Aub1 genomic profile, which undergoes dynamic changes during embryogenesis in both mice and *Drosophila* (Chen et al. 2021; Mei et al. 2021; Bonnet et al. 2022). During the onset of zygotic gene activation in *Drosophila* embryos, H2Aub1 is highly enriched at Polycomb target genes, where it promotes H3K27 trimethylation (Bonnet et al. 2022). At this stage, both modifications form block-like domains that closely mirror each other at these sites. In late-stage embryos and larvae, developmental stages during which the Polycomb machinery maintains repression of its target genes, H3K27me3 domains persist at Polycomb targets. Sustained H3K27me3 enrichment at these genes is essential to preserve long-term repression (Pengelly et al. 2013; McKay et al. 2015), as it marks local chromatin for interaction with the Pc subunit of cPRC1 (Messmer et al. 1992; Fischle et al. 2003; Min et al. 2003). In contrast, H2Aub1 is deubiquitinated by PR-DUB (Fig. 1A), reducing its enrichment to the uniformly low levels present throughout the rest of the genome (Bonnet et al. 2022). This removal is critical for effective repression, as H2Aub1 disrupts nucleosome stacking and is thought to antagonize cPRC1-mediated silencing through chromatin compaction (Francis et al. 2004; Bonnet et al. 2022). This in turn raises the question of which form of PRC2 sustains H3K27me3 enrichment at these loci to preserve Polycomb repression.

Here, we used mutants in genes encoding PRC1 or PRC2 subcomplex-specific subunits to assess how the different forms of these complexes shape the H2Aub1 and H3K27me3 genomic profiles in *Drosophila* embryos and evaluate the requirement of each complex for repression of classic Polycomb target genes. These analyses revealed both gene-specific and broad, genome-wide functions for the different complexes. Our investigation uncovered specific redundancies between complex subtypes, along with extensive cross-talk mediated by histone modifications. Together, these features endow the system with remarkably flexible buffering capacity, allowing the formation of functional Polycomb-repressed chromatin even under conditions of severe perturbation.

## Results

### vPRC1 generates the bulk of genome-wide H2Aub1, whereas cPRC1 deposits low levels of H2Aub1 solely at Polycomb target genes

To assess where and how cPRC1 and vPRC1 differ in their impact on H2A monoubiquitination in embryos, we deleted the E3 ligase activity of cPRC1, of vPRC1, or of all forms of PRC1. To eliminate vPRC1 activity, we generated a *l(3)73Ah*^*0*^ deletion allele that removes the coding region of *l(3)73Ah* (Supplemental Fig. S1A). *l(3)73Ah*^*0*^ mutant embryos that lacked both maternally deposited and zygotically expressed L(3)73Ah protein [*l(3)73Ah*^*0 m*−*z*−^] arrested development at the end of embryogenesis. To remove cPRC1 activity, we created a chromosomal deletion allele called *Psc-Su(z)2*^*0*^ by deleting the promoters and N-terminal coding regions of the juxtaposed *Psc* and *Su(z)2* genes (Supplemental Fig. S1B). Embryos lacking maternally deposited and zygotically expressed Psc and Su(z)2 proteins [*Psc-Su(z)2*^*0 m*−*z*−^] already arrested development during gastrulation, similar to mutants homozygous for *Su(z)2*^*1.b8*^, a larger chromosomal deletion that removes *Psc*, *Su(z)2*, and additional genes in the region (Gutiérrez et al. 2012). For this reason, we used *Psc-Su(z)2*^*0 m+z*−^ mutant embryos [called *Psc-Su(z)*2^*0*^] and focused our analyses for all genotypes on 21–24 h old embryos. To disable the E3 ligase activity of all forms of PRC1, we used Sce^I48A^ mutant embryos in which both the zygotically expressed and the maternally deposited Sce protein contained the Sce^Ile48Ala^ point mutation that abrogates E3 ligase activity for H2A monoubiquitination without disrupting cPRC1 or vPRC1 complex assembly (Pengelly et al. 2015; Bonnet et al. 2022).

Chromatin immunoprecipitation (ChIP) assays in 21–24 h old wild-type embryos showed that H2Aub1 was enriched at relatively uniform low levels across the entire genome (Fig. 1B [cf. tracks 1 and 2], C; cf. Bonnet et al. 2022). *l(3)73Ah*^*0*^ mutant embryos showed a drastic genome-wide reduction of H2Aub1 levels compared with wild-type embryos (Fig. 1B [cf. tracks 1 and 3], cf. C and D). In contrast, *Psc-Su(z)2*^*0*^ mutant embryos showed overall H2Aub1 enrichment levels similar to wild type (Fig. 1E). However, they also exhibited de novo enrichment of H2Aub1 in pronounced peaks at a subset of canonical Polycomb target genes, including *Ultrabithorax* (*Ubx*), *Antennapedia* (*Antp*), or *engrailed* (*en*) (Fig. 1B, cf. tracks 1 and 5; Supplemental Fig. S2). The molecular basis for this site-specific accumulation remains unclear.

Importantly, residual H2Aub1 ChIP signal persisted in *l(3)73Ah*^*0*^ mutants, whereas *Sce*^*I48A*^ mutants showed complete loss of H2Aub1 signal (Fig. 1B [cf. tracks 3,4 and 2], D; Supplemental Fig. S2A). The residual H2Aub1 enrichment in *l(3)73Ah*^*0*^ mutants closely mirrored domains with high H3K27me3 enrichment characteristic of Polycomb target genes (Fig. 1B, cf. tracks 3,4 and 6; Supplemental Fig. S2). Indeed, these regions all showed binding of cPRC1 (Fig. 1B, track 7; Supplemental Fig. S2), suggesting that the residual H2Aub1 enrichment in *l(3)73Ah*^*0*^ mutants was generated by cPRC1. Based on these criteria, we used chromosomal intervals marked by cPRC1-generated H2Aub1 and high H3K27me3 enrichment in *l(3)73Ah*^*0*^ mutant embryos (Supplemental Fig. S3) for genome segmentation with STAN to define canonical H3K27me3 domains (Materials and Methods). We conclude that vPRC1 generates the bulk of H2Aub1 across the entire genome, including at Polycomb target genes, and that cPRC1 deposits H2Aub1 solely at Polycomb targets but contributes less than vPRC1 to the H2Aub1 profile at these loci.

### Embryos lacking L(3)73Ah showed no homeotic phenotypes but arrested development at the end of embryogenesis

**Figure 2.**
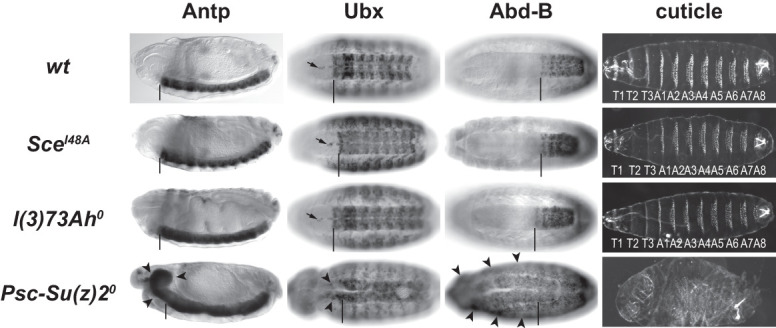
L(3)73Ah and H2Aub1 are required for viability but not for Polycomb repression of HOX genes. Side views (first column) and ventral views (second and third columns) of stage 15 or 16 embryos of the indicated genotypes stained with antibodies against Antp, Ubx, or Abd-B and ventral views (fourth column) of embryonic cuticles of the same genotypes. In images in the first three columns, the vertical bars mark the location of the normal anterior boundaries of the Antp, Ubx, and Abd-B expression domains; the small black arrows in the second column mark the Ubx-positive midline cells that are part of the normal Ubx expression pattern in wild type. Note that the Antp, Ubx, or Abd-B expression patterns and embryonic cuticles of *Sce*^*I48A*^ and *l(3)73Ah*^*0*^ mutants are indistinguishable from wild type. *Sce*^*I48A*^ and *l(3)73Ah*^*0*^ animals all arrested development at the end of embryogenesis (*N* > 150 for both genotypes). In *Psc-Su(z)2*^*0*^ embryos, all three HOX proteins are widely misexpressed (black arrowheads), and the cuticle shows highly abnormal morphology with poorly developed denticle bands. In all three mutant genotypes, the documented phenotypes were observed in 100% of the animals (*N* > 15 for all genotypes and stainings).

We next compared the phenotypes of *l(3)73Ah*^*0*^, *Psc-Su(z)2*^*0*^, and *Sce*^*I48A*^ mutant embryos. *l(3)73Ah*^*0*^ mutants all arrested development at the end of embryogenesis but showed no obvious morphological defects in the embryonic cuticle and normal expression of the HOX genes *Antennapedia* (*Antp*), *Ultrabithorax* (*Ubx*), and *Abdominal-B* (*Abd-B*). This phenotype closely resembled that of *Sce*^*I48A*^ mutants (Fig. 2; cf. Pengelly et al. 2015). In contrast, *Psc-Su(z)2*^*0*^ mutant embryos showed extensive misexpression of Antp, Ubx, and Abd-B and highly abnormal cuticle morphology with poorly developed denticle bands (Fig. 2), corroborating the observations made in *Su(z)2*^*1.b8*^ mutants in earlier studies (Soto et al. 1995; Gutiérrez et al. 2012). These observations show that L(3)73Ah, like Sce E3 ligase activity, is essential for development but dispensable for Polycomb repression at HOX genes. The severe Polycomb repression defects in *Psc-Su(z)2*^*0*^ mutants underscore the critical role of the nonenzymatic chromatin compaction activities of Psc and Su(z)2 for gene repression by cPRC1 (King et al. 2005).

### PRC2.1 and PRC2.2 together create the H3K27me3 landscape with genomic context-dependent contributions by each complex

We next assessed the relative contribution of PRC2.1 and PRC2.2 to the H3K27me3 profile. To remove PRC2.1 activity, we used embryos homozygous for a null mutation in *Polycomblike* (*Pcl*) (Nekrasov et al. 2007). For simplicity, we refer to the *Pcl*^*0 m+z*−^ mutant embryos used in our experiments as *Pcl*^*0*^ mutants. In the case of PRC2.2, it was technically not feasible to generate embryos that lacked both Jarid2 and Aebp2. As a proxy, we analyzed H3K27me3 in *Sce*^*I48Am*−*z*−^ mutant embryos in which Jarid2- and Aebp2-mediated PRC2.2 binding to H2Aub1-modified nucleosomes was eliminated due to the lack of H2Aub1. For comparison, we used *esc*^*0 m*−*z*−^ mutant embryos (referred to here as *esc*^*0*^) (Struhl 1981) that lacked the PRC2 core subunit Esc that is essential for HMTase activity in all forms of PRC2.

**Figure 3.**
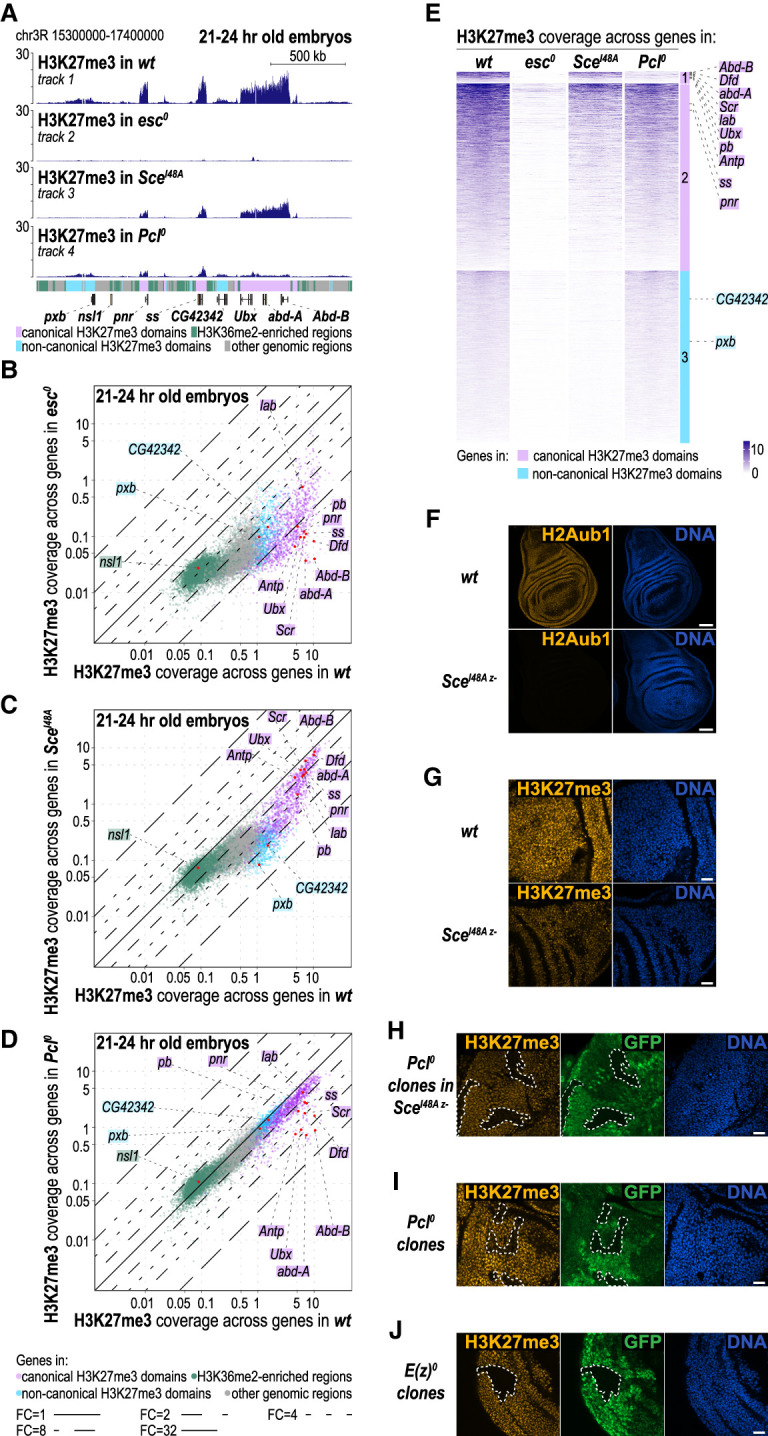
Redundant and unique contributions of PRC2.1 and PRC2.2 shape the H3K27me3 genomic profile. (*A*) H3K27me3 ChIP-seq profiles in 21–24 h old embryos of the indicated genotypes. Genomic interval is as in Figure 1B. (*B*) Scatter plots showing H3K27me3 read coverage across gene bodies in 21–24 h old wild-type (wt) and *esc*^*0*^ mutant embryos. Gene representation as dots and lines indicating fold changes are as in Figure 1. (*C*) Scatter plot as in *B* but comparing wild-type and *Sce*^*I48A*^ mutant embryos. (*D*) Scatter plot as in *B* but comparing wild-type and *Pcl*^*0*^ mutant embryos. (*E*) Heat map representation comparing H3K27me3 read coverage across gene bodies for all genes in canonical H3K27me3 domains (subclusters 1 and 2, marked by the pink bar) and noncanonical H3K27me3 domains (cluster 3, marked by the light blue bar) in the four indicated genotypes. Within each cluster, genes are ranked based on their average H3K27me3 read coverage in wild type. Genes in canonical H3K27me3 domains are represented as two subclusters: Subcluster 1 contains the *Antp-C* and *BX-C* genes where H3K27me3 reduction in *Pcl*^*0*^ mutants is almost as severe as in *esc*^*0*^ mutants, whereas subcluster 2 contains all other genes in canonical H3K27me3 domains. Also note that the H3K27me3 reduction of genes in cluster 3 in *Sce*^*I48A*^ mutants is nearly as severe as in *esc*^*0*^ mutants. The ranking positions of all genes shown in *A* and of the HOX genes from the *Antp-C* are indicated. (*F*) Wing imaginal discs from wild-type (wt) and *Sce*^*I48Az*−^ homozygous mutant larvae, stained with antibodies against H2Aub1 and Hoechst to visualize nuclei (DNA). Scale bar, 45 µm. (*G*) Wing imaginal discs from wild-type (wt) and *Sce*^*I48Az*−^ homozygous mutant larvae, stained with antibodies against H3K27me3 and Hoechst to visualize nuclei (DNA). Scale bar, 15 µm. (*H*) Eye imaginal disc from *Sce*^*I48A z*−^ homozygous mutant larva with clones of *Pcl*^*0*^ homozygous mutant cells, stained with antibody against H3K27me3. *Pcl*^*0*^ homozygous cells are marked by the absence of GFP (green), and the tissue containing *Sce*^*I48A*^ *Pcl*^*0*^ double-mutant cells is outlined with white dotted lines. Hoechst staining visualizes nuclei (DNA). Scale bar, 15 µm. Note the near-complete loss of H3K27me3 signal in *Sce*^*I48A*^ *Pcl*^*0*^ double-mutant cells. (*I*) Wing imaginal disc from *Pcl*^*0*^ heterozygous larva with clones of *Pcl*^*0*^ homozygous mutant cells, stained with antibody against H3K27me3. *Pcl*^*0*^ homozygous clone tissue is marked by the absence of GFP (green) and is outlined with white dotted lines as in *H*. Scale bar, 15 µm. (*J*) Wing imaginal disc from *E(z)*^*0*^ heterozygous larva with clones of *E(z)*^*0*^ homozygous mutant cells, stained with antibody against H3K27me3. *E(z)*^*0*^ homozygous clone tissue is marked by the absence of GFP (green) and is outlined with white dotted lines as in *H*. Scale bar, 15 µm.

In 21–24 h old wild-type embryos, ChIP for H3K27me3 enriched both canonical and noncanonical H3K27me3 domains. Noncanonical H3K27me3 domains differ from the above-defined canonical H3K27me3 domains in that they lack detectable PcG protein binding and show lower levels of H3K27me3 enrichment (Fig. 3A, track 1, colored bar below track 4; Bonnet et al. 2022).

As expected, 21–24 h old *esc*^*0*^ mutant embryos showed drastic loss of H3K27me3 genome-wide, similar to the reduction previously reported in early-stage *esc*^*0*^ mutant embryos (Fig. 3A [cf. tracks 2 and 1], B; cf. Bonnet et al. 2019). The residual H3K27me3 signal in *esc*^*0*^ mutant embryos likely reflects H3K27me3 deposition by PRC2 containing the Esc paralog Esc-like (Escl), which showed upregulated expression in *esc*^*0*^ mutants (Ohno et al. 2008).

In *Sce*^*I48A*^ mutants, noncanonical H3K27me3 domains showed strongly reduced H3K27me3 enrichment, reminiscent of *esc*^*0*^ animals (Fig. 3A [cf. tracks 3 and 1], cf. B and C; Supplemental Fig. S3A; cf. Bonnet et al. 2022). *Sce*^*I48A*^ mutants also showed reduced H3K27me3 levels at canonical H3K27me3 domains, but the reduction was less pronounced than in *esc*^*0*^ mutant embryos Fig. 3C). At HOX genes, H3K27me3 levels were reduced by less than twofold compared with wild type (Fig. 3A [cf. tracks 3 and 1], C; Supplemental Fig. S3A; cf. Bonnet et al. 2022).

Analysis of H3K27me3 profiles in *l(3)73Ah*^*0*^ or *Psc-Su(z)2*^*0*^ mutants suggested that formation of noncanonical H3K27me3 domains was exclusively driven by vPRC1-generated H2Aub1 (Supplemental Fig. S3, cf. B and C). At genes in canonical H3K27me3 domains, the relative contribution of vPRC1 and cPRC1 seemed to vary considerably between genes (Supplemental Fig. S3), but the changes in the H3K27me3 profile in *Psc-Su(z)2*^*0*^ mutants should be interpreted in light of potential secondary effects arising from widespread target gene deregulation in these mutants (Fig. 2; see Supplemental Fig. S3 legend for extended discussion).

In *Pcl*^*0*^ mutant embryos, only genes in canonical but not in noncanonical H3K27me3 domains showed reduced H3K27me3 enrichment. At most of the affected genes, however, the H3K27me3 reduction was less than twofold, and only a small subset of genes showed a reduction greater than fourfold (Fig. 3A [cf. tracks 4 and 1], D). Of note, the genes with the most severely reduced H3K27me3 levels included almost all of the HOX genes in the Antennapedia and Bithorax complexes (Fig. 3D,E).

Notably, none of the genes in canonical H3K27me3 domains exhibited a loss of H3K27me3 in *Pcl*^*0*^ or *Sce*^*I48A*^ mutants that was as severe as in *esc*^*0*^ mutant embryos (Fig. 3, cf. C,D and B). This prompted us to assess H3K27me3 levels in animals with *Pcl*^*0*^ *Sce*^*I48A*^ double-mutant cells. As it was not possible to generate sufficient quantities of *Pcl*^*0*^ *Sce*^*I48A*^ double-mutant embryos for ChIP-seq analysis, we analyzed H3K27me3 bulk levels by immunofluorescence labeling. We had previously found that the presence of maternally deposited wild-type Sce protein in early embryos permitted *Sce*^*I48A*^ zygotic mutant animals (*Sce*^*I48A z*−^) to develop up to the pupal stage (Pengelly et al. 2015). Immunofluorescence labeling of imaginal discs from *Sce*^*I48A z*−^ mutant larvae showed that these tissues lacked detectable H2Aub1 (Fig. 3F) and that H3K27me3 bulk levels were considerably reduced (Fig. 3G), consistent with the observations by ChIP-seq in embryos (Fig. 3C). Clones of *Pcl*^*0*^ homozygous cells induced in *Sce*^*I48A z*−^ mutant animals analyzed 72 h after clone induction showed a drastic reduction of H3K27me3 signal (Fig. 3H) that was much more severe than in the neighboring *Sce*^*I48A z*−^ single-mutant cells or in clones of *Pcl*^*0*^ single-mutant cells (Fig. 3I). The *Pcl*^*0*^ *Sce*^*I48A*^ double-mutant cells still retained very weak nuclear H3K27me3 signal (Fig. 3H), whereas cell clones lacking the PRC2 catalytic subunit E(z) show a complete loss of H3K27me3 (Fig. 3J). In conclusion, simultaneous removal of the PRC2.1 accessory factor Pcl and of the H2Aub1 docking site for PRC2.2 on nucleosomes almost completely eliminated H3K27me3 deposition by PRC2.

Together, our data suggest that most H3K27me3 domains are formed by the combined action of PRC2.1 and PRC2.2, but the relative contribution of each complex depends on the genomic context. PRC2.2, via interaction with H2Aub1, is the main enzyme that creates noncanonical H3K27me3 domains. PRC2.1 acts predominantly at canonical H3K27me3 domains, but its activity is limiting only at HOX and a few other Polycomb target genes.

### Increased H2Aub1 levels at Polycomb targets enable PRC2.2 to restore functional H3K27me3 domains in the absence of PRC2.1

**Figure 4.**
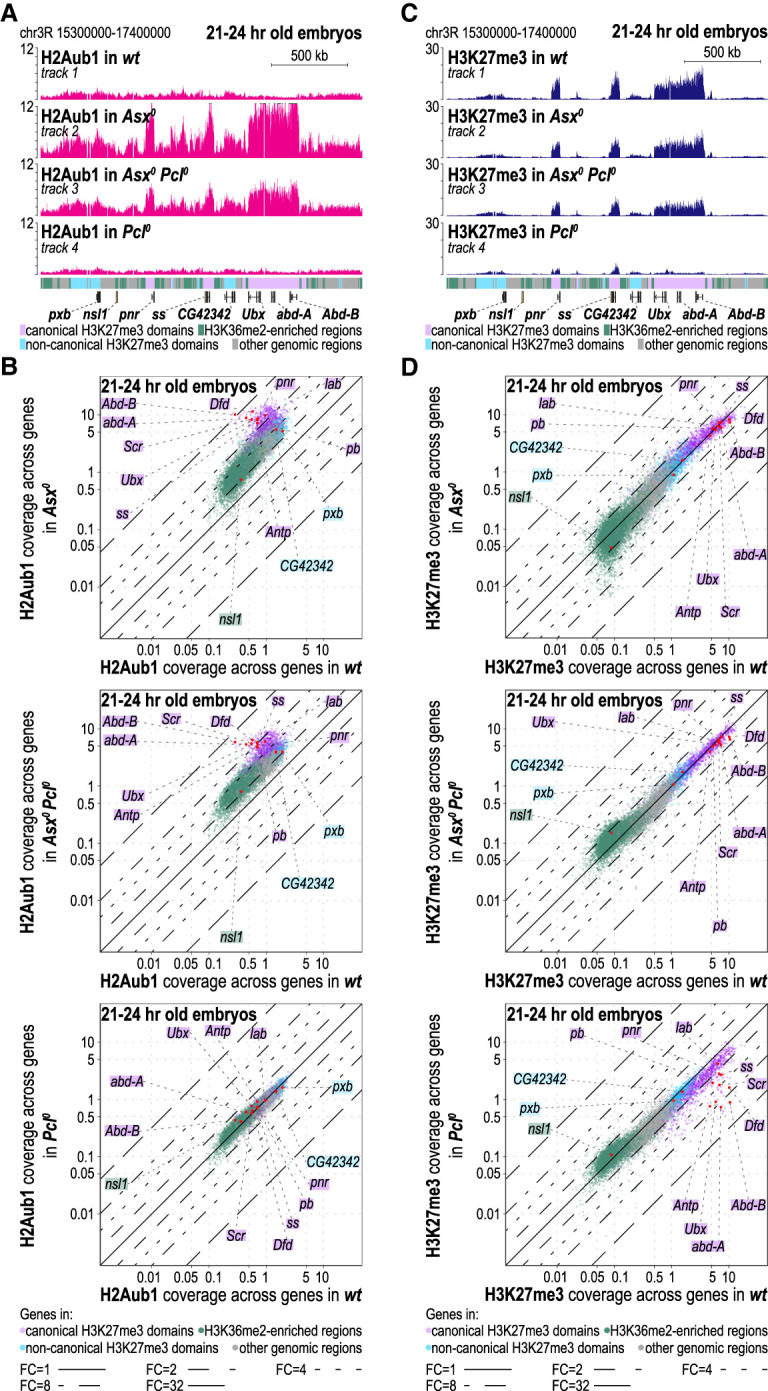
Increased H2Aub1 levels at Polycomb target genes in PR-DUB mutants enable PRC2.2 to restore functional H3K27me3 domains when PRC2.1 is absent. (*A*) H2Aub1 ChIP-seq profiles in 21–24 h old embryos of the indicated genotypes. Genomic interval are as in Figure 1B. Note the extensive increase in H2Aub1 levels at genes in canonical H3K27me3 domains in *Asx*^*0*^ single mutants and *Asx*^*0*^ *Pcl*^*0*^ double mutants. (*B*) Scatter plots showing H2Aub1 read coverage across gene bodies in 21–24 h old embryos, comparing wild-type (wt) with *Asx*^*0*^ single-mutant (*top* panel), wt with *Asx*^*0*^ *Pcl*^*0*^ double-mutant (*middle* panel), or wt with *Pcl*^*0*^ single-mutant (*bottom* panel) embryos. Layout is as in Figure 1C. (*C*) As in *A* but showing the H3K27me3 ChIP-seq profiles in the same genotypes. Note the severe reduction of H3K27me3 at canonical H3K27me3 domains in *Pcl*^*0*^ single mutants and the restoration of high H3K27me3 levels in these domains in *Asx*^*0*^ *Pcl*^*0*^ double mutants. (*D*) As in *B* but with H3K27me3 read coverage plotted.

In embryos lacking PR-DUB (i.e., in *Asx*^*0*^ mutants), H2Aub1 levels across the genome were about fourfold higher than in wild type, and at HOX and other Polycomb targets, the increase was even 20-fold to 30-fold, as reported previously (Fig. 4A [cf. tracks 2 and 1], B; cf. Bonnet et al. 2022). In contrast, the H3K27me3 profile in these mutants was largely unchanged compared with wild type (Fig. 4C [cf. tracks 2 and 1], D; cf. Bonnet et al. 2022). Considering that the majority of nucleosomes in canonical H3K27me3 domains appear to be trimethylated at H3K27 (Bonnet et al. 2019), it is expected that an increase in H2Aub1-modified nucleosomes in these regions would not lead to additional H3K27 trimethylation by PRC2.2. We therefore next tested whether increased H2Aub1 coverage would be able to promote H3K27me3 deposition by PRC2.2 in animals in which H3K27me3 had been depleted by removal of PRC2.1. Strikingly, in *Asx*^*0*^ *Pcl*^*0*^ double mutants, HOX and other Polycomb target genes no longer showed the drastic reduction in H3K27me3 levels that occurs in *Pcl*^*0*^ single mutants; instead, the double mutants had substantially restored H3K27me3 domains that resembled those in wild type (Fig. 4C [cf. tracks 3 and 4], D). This suggests that in animals lacking both PRC2.1 and PR-DUB, the massive increase in H2Aub1 levels at Polycomb target genes (Fig. 4A [track 3], B) allowed PRC2.2 to restore a near-regular H3K27me3 profile at target genes where H3K27me3 deposition normally relies on PRC2.1 (Fig. 4C [cf. tracks 3 and 4], D).

**Figure 5.**
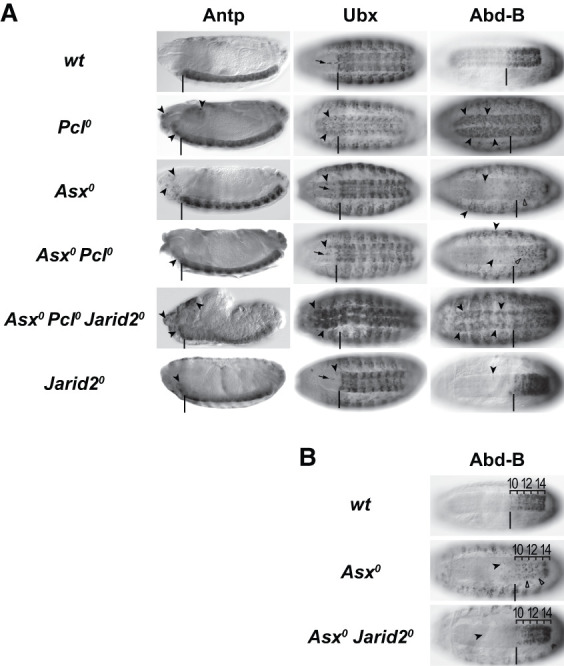
Impacts of the H2Aub1–PRC2.2 feedback loop on Polycomb repression at HOX genes. (*A*) Polycomb repression defects in the absence of PRC2.1 are rescued in PRC2.1, PR-DUB double mutants due to compensatory H3K27me3 deposition by PRC2.2. Side views (first column) and ventral views (second and third columns) of stage 15 or 16 embryos of the indicated genotypes, stained with antibodies against Antp, Ubx, or Abd-B. The vertical bars mark the locations of the normal anterior boundaries of the Antp, Ubx, and Abd-B expression domains; the small black arrows in the second column mark Ubx-positive midline cells that are part of the normal Ubx expression pattern in wild type. Black arrowheads mark misexpression, and the empty arrowheads mark loss of expression. *Pcl*^*0*^ mutants show extensive misexpression of all three HOX genes, most notably in the central nervous system (CNS). In *Asx*^*0*^ mutants, only a few scattered cells in the CNS show HOX gene misexpression; only Abd-B is widely misexpressed in the epidermis. The loss of Abd-B expression in CNS cells (empty arrowheads) is discussed in *B*. In *Asx*^*0*^ *Pcl*^*0*^ double mutants, the HOX gene misexpression observed in the CNS of *Pcl*^*0*^ single mutants is largely suppressed, and the residual misexpression resembles that in *Asx*^*0*^ single mutants. This suppression is lost in *Asx*^*0*^ *Pcl*^*0*^ *Jarid2*^*0*^ mutants where all three HOX genes are widely misexpressed. *Jarid2*^*0*^ single-mutant embryos show ectopic expression of HOX genes only in a few scattered cells; these embryos arrest development at the end of embryogenesis and fail to hatch from the eggshell. The phenotypes shown in all mutant genotypes were observed in 100% of the animals (*N* > 8 for all genotypes). (*B*) The trithorax-like loss of *Abd-B* expression in the CNS of PR-DUB mutant embryos is due to ectopic repression by PRC2.2. Ventral views of stage 16 embryos, stained with antibodies against Abd-B. Labeling is as in *A*; in addition, the positions of parasegments (PSs) 10–14 are indicated. Note that the loss of Abd-B expression in the CNS in PS10–PS13 (empty arrowheads) in *Asx*^*0*^ mutants (see also in the *Asx*^*0*^ embryo in *A*) is partially suppressed in *Asx*^*0*^ *Jarid2*^*0*^ double mutants.

The restored H3K27me3 profile at HOX genes in *Asx*^*0*^ *Pcl*^*0*^ double mutants also mitigated the defects in HOX gene repression observed in *Pcl*^*0*^ single-mutant embryos (Fig. 5A). *Asx*^*0*^ *Pcl*^*0*^ double mutants no longer showed the widespread misexpression of *Antp*, *Ubx*, and *Abd-B* that is observed in the central nervous system (CNS) of *Pcl*^*0*^ single mutants (Fig. 5A). The remaining repression defects in *Asx*^*0*^ *Pcl*^*0*^ double mutants closely resembled those in *Asx*^*0*^ single mutants, where the three HOX genes were misexpressed mainly in the epidermis and only in a few rare cells in the CNS (Fig. 5A). These repression defects in *Asx*^*0*^ mutants are thought to result from defects in the structural organization of chromatin at these genes due to excessive H2Aub1 accumulation (Bonnet et al. 2022).

Importantly, the restored HOX gene repression in *Asx*^*0*^ *Pcl*^*0*^ double mutants was abolished when the Jarid2-mediated interaction of PRC2.2 with H2Aub1 was eliminated. Specifically, we removed maternally deposited and zygotically expressed Jarid2 protein to generate *Asx*^*0*^ *Pcl*^*0*^ *Jarid2*^*0*^ triple mutant animals, and these showed widespread misexpression of all three HOX genes (Fig. 5A). A previously described *Jarid2^0^* deletion allele (Shalaby et al. 2017) was used for these experiments. In *Jarid2*^*0*^ single-mutant control embryos, expression of Antp, Ubx, and Abd-B was overall confined to their normal expression domains, with only a few rare scattered cells showing misexpression (Fig. 5A). Moreover, in *Jarid2*^*0*^ single mutants, H3K27me3 levels were undiminished or less than twofold reduced in canonical H3K27me3 domains and were more substantially reduced in only a subset of noncanonical H3K27me3 domains (Supplemental Fig. S4). Together, this further corroborates the notion that the increased H2Aub1 enrichment in *Asx*^*0*^ *Pcl*^*0*^ double mutants enables PRC2.2 to build functional H3K27me3 domains at HOX and other Polycomb target genes and that PRC2.2 thereby effectively compensates for the lack of PRC2.1 activity.

### The trithorax-like loss of Abd-B expression in PR-DUB mutants is due to faulty H3K27me3 deposition by PRC2.2

In addition to misexpression of Antp, Ubx, and Abd-B, embryos lacking PR-DUB also showed contextual loss of expression of Abd-B. Specifically, although cells in the epidermis and mesoderm of *Asx* mutant embryos exhibit ectopic expression of *Abd-B* anterior to parasegment (PS) 10, many cells in the central nervous system within PS10–PS13 displayed a marked loss of *Abd-B* expression—resembling the phenotype observed in trithorax group mutants (Fig. 5B; cf. Scheuermann et al. 2010; Bonnet et al. 2022). In wild-type embryos, Abd-B is expressed in a step-like pattern in the epidermis and CNS, with the lowest levels in PS10 and the highest levels in PS14 (Fig. 5B; cf. Celniker et al. 1990). This graded expression is generated by PS-specific enhancers in the 3′ *cis*-regulatory region of *Abd-B*, and there is compelling genetic evidence that expression in PS10–PS13 is modulated by H3K27me3 levels. First, removal of PRC2 activity [i.e., in *esc* or *E(z)* mutants] resulted in PS14-like high-level expression of Abd-B along the entire body axis (Simon et al. 1992), arguing that the presence of H3K27me3 normally dampens expression in PS10–PS13. Second, removal of the H3K27me3 demethylase Utx resulted in complete loss of Abd-B expression in PS10 and reduced expression also in cells in more posterior PSs (Copur and Müller 2013), suggesting that in wild-type animals, Utx counteracts excessive H3K27me3 deposition by PRC2 and thereby prevents unwanted installation of full Polycomb repression in cells where Abd-B needs to be expressed.

The loss of Abd-B expression in the CNS of *Asx*^*0*^ mutant embryos similarly appears to result from excessive H3K27me3 deposition by PRC2.2, promoted by the massive accumulation of H2Aub1 at Abd-B in the absence of PR-DUB activity. *Asx*^*0*^ *Jarid2*^*0*^ double-mutant embryos showed a substantially restored Abd-B expression pattern in CNS cells in PS10–PS13 (Fig. 5B).

## Discussion

In this study, we investigated the locus-specific and global roles of the different subtypes of PRC1 and PRC2 in shaping the H2Aub1 and H3K27me3 genomic landscapes in developing *Drosophila*. These analyses identified unique as well as redundant functions of paralogous Polycomb complexes but also uncovered that the system has a remarkably flexible compensatory capacity for forming functional chromatin domains.

### vPRC1 and cPRC1 make distinct contributions to shaping the H2Aub1 profile

We found that L(3)73Ah-containing vPRC1 complexes account for the predominant portion of the genome-wide H2Aub1 profile in embryos. L(3)73Ah (the single vPRC1-specific PCGF protein in *Drosophila*) and Sce have been proposed to form the core of two distinct forms of vPRC1 in *Drosophila* (Fig. 1A; Kang et al. 2022). Although the removal of different PCGF proteins in mouse embryonic stem cells suggested that different vPRC1 complexes contribute in distinct ways to the genomic H2Aub1 profile (vPRC1.1 is primarily responsible for the strong enrichment at CpG island promoters, whereas vPRC1.3 and vPRC1.5 support the broader, low-level genome-wide H2Aub1 profile) (Fursova et al. 2019), our experiments in L(3)73Ah mutants did not allow us to distinguish which of the two forms of vPRC1 forms deposits the bulk of H2Aub1 in flies. However, considering that <3% of total H2A is monoubiquitinated at K118 in late-stage wild-type *Drosophila* embryos (Scheuermann et al. 2010), it would appear that the responsible vPRC1 complexes act in an untargeted fashion to monoubiquitinate H2A in a stochastic manner along the entire genome. In contrast, cPRC1 appears to selectively catalyze H2A monoubiquitination only at Polycomb target genes, where cPRC1 association is readily detected by ChIP (Fig. 1; Supplemental Fig. S2).

Two further points deserve consideration. First, in contrast to late-stage embryos, where H2Aub1 is distributed at relatively uniform low levels across the genome, blastoderm stage embryos exhibit block-like H2Aub1 enrichment domains that coextend with both canonical and noncanonical H3K27me3 regions. (Bonnet et al. 2022). Second, in PR-DUB mutants, these block-like H2Aub1 domains persist at these same regions also in late-stage embryos (Fig. 4). Which form of PRC1 creates these H2Aub1 domains? It has not been possible to generate sufficient quantities of blastoderm stage *l(3)73Ah*^*0 m*−*z*−^ mutant embryos or of PR-DUB mutant embryos that simultaneously also lacked vPRC1 activity [e.g., *Asx*^*z–*^*l(3)73Ah*^*0 m*−*z*−^ double mutants] for ChIP-seq analyses. We hypothesize that in both instances (i.e., blastoderm stage wild-type and late-stage PR-DUB mutant embryos), it is the combined E3 ligase activities of both vPRC1 and cPRC1 that create these H2Aub1 domains.

Moreover, H2Aub1 levels in PR-DUB mutant embryos are elevated not only at Polycomb target genes but, at lower levels, also globally across the genome (Fig. 4; Bonnet et al. 2022). We propose that vPRC1 catalyzes this untargeted genome-wide H2A monoubiquitination.

Finally, we note that *Sce*^*I48A*^ and *l(3)73Ah*^*0*^ mutant embryos both arrested development at the end of embryogenesis but showed no gross morphological abnormalities or deregulation of HOX gene expression (Fig. 2; Pengelly et al. 2015). Both mutants showed a highly similar near-complete loss of H3K27me3 at noncanonical H3K27me3 domains (Supplemental Fig. S3). However, it should be noted that RNA-seq analyses in *Sce*^*I48A*^ mutant embryos had not detected a systematic deregulation of genes in noncanonical or canonical H3K27me3 domains (Bonnet et al. 2022). Further studies will thus be necessary to clarify how alterations in H2Aub1 and H3K27me3 profiles relate to transcriptional changes and contribute to the developmental arrest observed in *Sce*^*I48A*^ and *l(3)73Ah*^*0*^ mutants.

### Redundant and genomic context-specific contributions by PRC2.1 and PRC2.2 shape canonical and noncanonical H3K27me3 domains

Genetic ablation of Pcl directly eliminated the formation of PRC2.1, whereas the removal of H2Aub1 in *Sce*^*I48A*^ mutants did not perturb PRC2.2 complex assembly but instead disrupted the interaction of the complex with H2Aub1 nucleosomes. Nevertheless, we found that in cells lacking both Pcl and H2Aub1 (i.e., in *Pcl*^*0*^ *Sce*^*I48A*^ double-mutant cells), H3K27 trimethylation was almost completely eliminated (Fig. 3F–I). This not only illustrates that it is the combined activity of PRC2.1 and PRC2.2 that generates the H3K27me3 genomic landscape but also suggests that PRC2.2 is primarily targeted to chromatin through interaction with H2Aub1-modified nucleosomes.

In *Sce*^*I48A*^ mutants, H3K27me3 levels at noncanonical H3K27me3 domains were almost as drastically reduced as in *esc*^*0*^ mutants (Fig. 3B,C,E), suggesting that PRC2.2 is the main enzyme responsible for generating the H3K27me3 mark at noncanonical domains. Considering that H2Aub1 is only present at low levels in these regions in late-stage embryos, this may appear paradoxical. A plausible explanation is that the H3K27me3 nucleosomes at noncanonical H3K27me3 domains in late-stage embryos were modified by PRC2.2 earlier during embryogenesis when, as discussed above, these domains were decorated with high levels of H2Aub1 (Bonnet et al. 2022). Because the majority of cells in mid- to late-stage embryos do not undergo cell divisions, such H3K27me3-modified nucleosomes would be expected to persist in these regions.

Canonical H3K27me3 domains showed a more moderate reduction of H3K27me3 levels in *Sce*^*I48A*^ single mutants (Fig. 3C). Moreover, with the exception of HOX genes, these regions also showed only a mild reduction of H3K27me3 levels in *Pcl*^*0*^ single mutants (Fig. 3D). Most canonical H3K27me3 domains are therefore generated by the combined action of PRC2.1 and PRC2.2, with the two complexes acting redundantly. This is strikingly reminiscent of the situation in embryonic stem cells, where at the majority of PcG target genes, PRC2.1 and PRC2.2 also act synergistically to generate H3K27me3 domains (Healy et al. 2019; Højfeldt et al. 2019).

The deregulation of HOX gene expression mutants in relation to changes in H3K27me3 levels at these genes allowed us to more finely correlate H3K27me3 levels with Polycomb repression. In *Sce*^*I48A*^ mutants, H3K27me3 levels at the HOX genes *Antp*, *Ubx*, and *Abd-B* were about twofold reduced, yet Polycomb repression at these genes appeared unperturbed. In contrast, in *Pcl*^*0*^ mutants, H3K27me3 levels at these three genes were about eightfold reduced compared with wild type, and all three genes were misexpressed. At least at these three HOX genes, the system therefore appears to be buffered to tolerate a twofold reduction in H3K27me3 coverage, whereas a more extensive reduction of H3K27me3 results in the breakdown of Polycomb repression.

### Compensatory H3K27 trimethylation by PRC2.2 through cross-talk with H2Aub1

The rescue of H3K27me3 domains at canonical Polycomb target genes in *Asx*^*0*^ *Pcl*^*0*^ double mutants but not in *Pcl*^*0*^ single mutants suggested that the high H2Aub1 accumulation at these Polycomb targets accounted for restored H3K27 trimethylation by PRC2.2. Genome-wide profiling of PRC2 core subunits revealed that the complex binds in a sharply localized manner to Polycomb response elements (PREs)—nucleosome-depleted *cis*-regulatory DNA sequences within canonical H3K27me3 domains where recruitment is driven by sequence-specific DNA-binding proteins (Brown et al. 1998; Kahn et al. 2006; Mohd-Sarip et al. 2006; Papp and Müller 2006; Schwartz et al. 2006; Herz et al. 2012; Frey et al. 2016). In contrast, ChIP-seq failed to detect PRC2 at the surrounding chromatin within these domains, at noncanonical H3K27me3 domains, or elsewhere in the genome. This is perhaps to be expected, considering that late-stage embryos contain only ∼2000 copies of PRC2 molecules per nucleus and that the complex, in addition to creating H3K27me3 chromatin, also catalyzes H3K27 monomethylation and dimethylation on 70%–80% of total histone H3 (Bonnet et al. 2019). Beyond its stable binding at PREs, the association of PRC2.1 and PRC2.2 with chromatin therefore is highly dynamic. In vitro studies on PRC2.1 found that DNA binding by Pcl prolongs PRC2 residence time on chromatin and thereby promotes H3K27 trimethylation (Choi et al. 2017). We envisage that the very high abundance of H2Aub1-modified nucleosomes at Polycomb targets in *Asx*^*0*^ *Pcl*^*0*^ double-mutant embryos functions via a similar principle: Jarid2/Aebp2-mediated H2Aub1 recognition simply prolongs the residence time of otherwise highly mobile PRC2.2 at the chromatin of these genes (Kalb et al. 2014; Cooper et al. 2016; Kasinath et al. 2021). This interaction, together with the allosteric activation of PRC2.2 through interaction of Esc with H3K27me3 in newly methylated nucleosomes (Hansen et al. 2008; Margueron et al. 2009; Jiao and Liu 2015; Oksuz et al. 2018), would thus enable efficient formation of canonical H3K27me3 domains in the absence of PRC2.1.

In conclusion, histone modification cross-talk that enables alternative chromatin targeting pathways equips the Polycomb system with a compensatory capacity to create functional chromatin domains. Such flexibility and adaptability may be especially critical during developmental transitions when chromatin must rapidly undergo dynamic changes. Conversely, this plasticity might also be harnessed to develop therapies for congenital diseases or cancers associated with mutations in Polycomb group regulators.

## Materials and methods

### Generation of the *l(3)73Ah*^*0*^ and *Psc-Su(z)2*^*0*^ deletion alleles

The *l(3)73Ah*^*0*^ allele was generated by imprecise excision of a P{PZ} transposable element inserted in the 5′ UTR of *l(3)73Ah* (Bloomington *Drosophila* Stock Center 11571). The excised chromosome was crossed to this original *l(3)73Ah* P{PZ} allele to check for lethality. The genomic sequence of the *l(3)73Ah*^*0*^ allele is shown in Supplemental Figure S1A. The *Psc-Su(z)2*^*0*^ short chromosomal deletion allele was generated by CRISPR–Cas9 technology. The genomic sequence of the engineered allele is shown in Supplemental Figure S1B.

### *Drosophila* strains and genotypes

See Supplemental Table S1 for detailed genotypes used in every experiment.

### Antibodies

Antibodies used in this study are listed in Supplemental Table S2.

### Embryo collection, chromatin preparation, and ChIP

Wild-type embryos (21–24 h old) and mutant embryos of the same developmental stage—*Sce*^*I48A*^, *l(3)73Ah*^*0*^, *Psc-Su(z)2*^*0*^, *esc*^*0*^, *Pcl*^*0*^, *Asx*^*0*^, and *Asx*^*0*^ *Pcl*^*0*^ (see Supplemental Table S1 for details on the genotypes)—were dechorionated, flash-frozen in liquid nitrogen, and stored at −80°C. Chromatin was prepared as described previously (Finogenova et al. 2020), and 500 ng of chromatin was used for each ChIP experiment. To enable subsequent normalization of the different ChIP-seq data sets, 100 ng of independently prepared *Drosophila pseudoobscura* chromatin was spiked into each sample prior to antibody addition. ChIP was then performed following the protocol described previously (Bonnet et al. 2019).

### Library preparation and sequencing

Library preparation was performed according to the manufacturer's instructions, followed by paired-end DNA sequencing (see the GEO entry for detailed information). Sequencing reads were aligned using STAR (Dobin et al. 2013) to both the *D. melanogaster* dm6 genome assembly (dos Santos et al. 2015) and the *D. pseudoobscura* dp3 genome assembly (FlyBase release 1.03, November 2004). Only reads that mapped uniquely to the genome, allowing a maximum of two mismatches, were retained for downstream analyses.

### Normalization of ChIP-seq data sets

For histone mark ChIP-seq data sets, normalization was performed using the proportion of *D. pseudoobscura* reads to *D. melanogaster* reads in both input and ChIP samples; for details, see Supplementary File 2 of Finogenova et al. (2020). For ChIP experiments using antibodies against Polycomb group proteins, normalization was based on the total number of mapped reads in each data set.

### Identification of H3K36me2- and H3K27me3-enriched regions

H3K36me2-enriched regions, H3K27me3-enriched regions (encompassing both canonical and noncanonical H3K27me3 domains), and other genomic regions were originally defined in an earlier work (Bonnet et al. 2022). In this study, the method for subdividing H3K27me3-enriched regions into canonical and noncanonical domains was refined. Canonical H3K27me3 domains were defined as regions exhibiting both H3K27me3 ChIP-seq signal and significant residual H2Aub1 ChIP signal in *l(3)73Ah*^*0*^ mutant embryos. This analysis was performed using the Bioconductor STAN package (Zacher et al. 2017) using a Poisson log normal distribution. Hidden Markov models were fitted with a maximum of 100 iterations.

### Calculation of read coverage on gene bodies

ChIP-seq read coverage across gene bodies was calculated for genomic intervals spanning from 750 bp upstream of transcription start sites to 750 bp downstream from transcription termination sites. Read coverage was defined as the ratio of the normalized number of mapped reads per million (RPM) from a ChIP-seq data set to the RPM from the corresponding input data set within the same genomic region. Among *D. melanogaster* FlyBase genes, 9952 were located in H3K36me2-enriched regions, 1162 were located in canonical H3K27me3 domains, 1006 were located in noncanonical H3K27me3 domains, and 5609 were located in other genomic regions, based on data from 21–24 h old embryos. A gene was assigned to a canonical or noncanonical H3K27me3 domain if at least 80% of its gene body interval overlapped with the respective domain.

### Immunohistochemistry and immunofluorescence stainings

Embryos of the appropriate genotypes (Supplemental Table S1) were fixed and stained with Antp, Ubx, and Abd-B antibodies following standard protocols. Imaginal discs from third instar larvae were stained with H3K27me3 or H2Aub1 and GFP as primary antibodies and Cy3- and Alexa 488-labeled secondary antibodies following standard protocols. Embryos devoid of both maternally deposited and zygotically expressed L(3)73Ah or Jarid2 were generated with the ovoD female sterile technique (Chou and Perrimon 1992). Embryos in which all maternally deposited and zygotically expressed Sce protein contained the Sce^Ile48Ala^ mutation were generated as described previously (Pengelly et al. 2015). For clonal analysis in larvae, clones were induced by heat-shock-induced expression of Flp recombinase in the genotypes listed in Supplemental Table S1. In Supplemental Figure S3, F and G, tissues dissected from wild-type and *Sce*^*I48A*^ mutant larvae were stained in the same tube using anti-H2Aub1 and H3K27me3 antibodies as indicated, and a GFP marker gene present in wild-type larvae was used to distinguish between the two genotypes.

### Data availability

Genomic data have been deposited in GEO under accession number GSE223899. The data sets used for each figure panel are listed in Supplemental Table S3.

## Supplemental Material

Supplement 1

Supplement 2

Supplement 3

Supplement 4
