# Supplementary material for: Histone modification cross-talk and protein complex diversification confer plasticity to Polycomb repression
Source: Genes Dev. 2026 Jan 1;40(1-2):43–55. doi: 10.1101/gad.353148.125 (PMC12758141; doi:10.1101/gad.353148.125)
Supplement: Supplement 1 [file Supplemenal_Material.pdf]

Figure S1

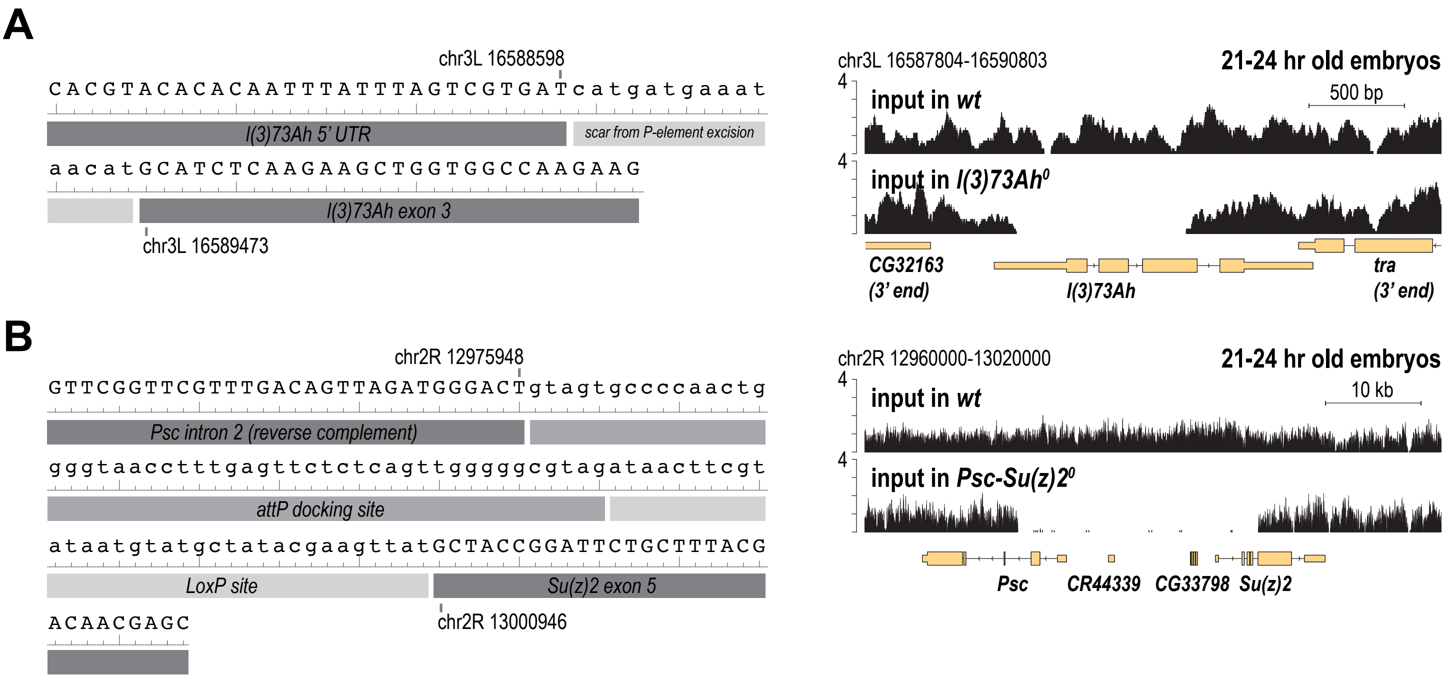
A
chr3L 16588598
CACGTACACACAATTTATTTAGTCGTGATcatgatgaaat
l(3)73Ah 5' UTR
scar from P-element excision
aacatGCATCTCAAGAAGCTGGTGGCCAAGAAG
l(3)73Ah exon 3
chr3L 16589473
chr3L 16587804-16590803
21-24 hr old embryos
4
input in wt
500 bp
4
input in l(3)73Ah0
CG32163
(3' end)
l(3)73Ah
tra
(3' end)
B
chr2R 12975948
GTTCGGTTCGTTTGACAGTTAGATGGGACTgtagtgccccaactg
Psc intron 2 (reverse complement)
gggtaacctttgagttctctcagttgggggcgtagataacttcgt
attP docking site
ataatgtatgctatacgaagttatGCTACCGGATTCTGCTTTACG
LoxP site
Su(z)2 exon 5
chr2R 13000946
ACAACGAGC
chr2R 12960000-13020000
21-24 hr old embryos
4
input in wt
10 kb
4
input in Psc-Su(z)20
Psc
CR44339
CG33798
Su(z)2

Figure S1

Genomic sequences of *l(3)73Ah^0* and *Psc-Su(z)2^0* alleles

(A) Left: genomic sequence (chr3L 16588570-16589500) flanking the deletion (chr3L 16588599-16589472) in the *l(3)73Ah^0* allele. The imperfect excision of the P-element from the parental line (see Materials and Methods) resulted in a scar of 16 bp, as indicated. Right: input profiles from *wt* and *l(3)73Ah^0* chromatins showing deletion of the three first *l(3)73Ah* exons in the mutant allele.

(B) Left: genomic sequence (chr2R 12975919-13000975) surrounding the deleted region in the *Psc-Su(z)2^0* allele. The genomic region from chr2R 12975949 to 13000945 was replaced by an *attP* docking site and a *LoxP* site, as indicated. Right: input profiles from *wt* and *Psc-Su(z)2^0* chromatins indicating deletion of *Psc* and *Su(z)2* promoters and 5' exons in the mutant allele.

Figure S2

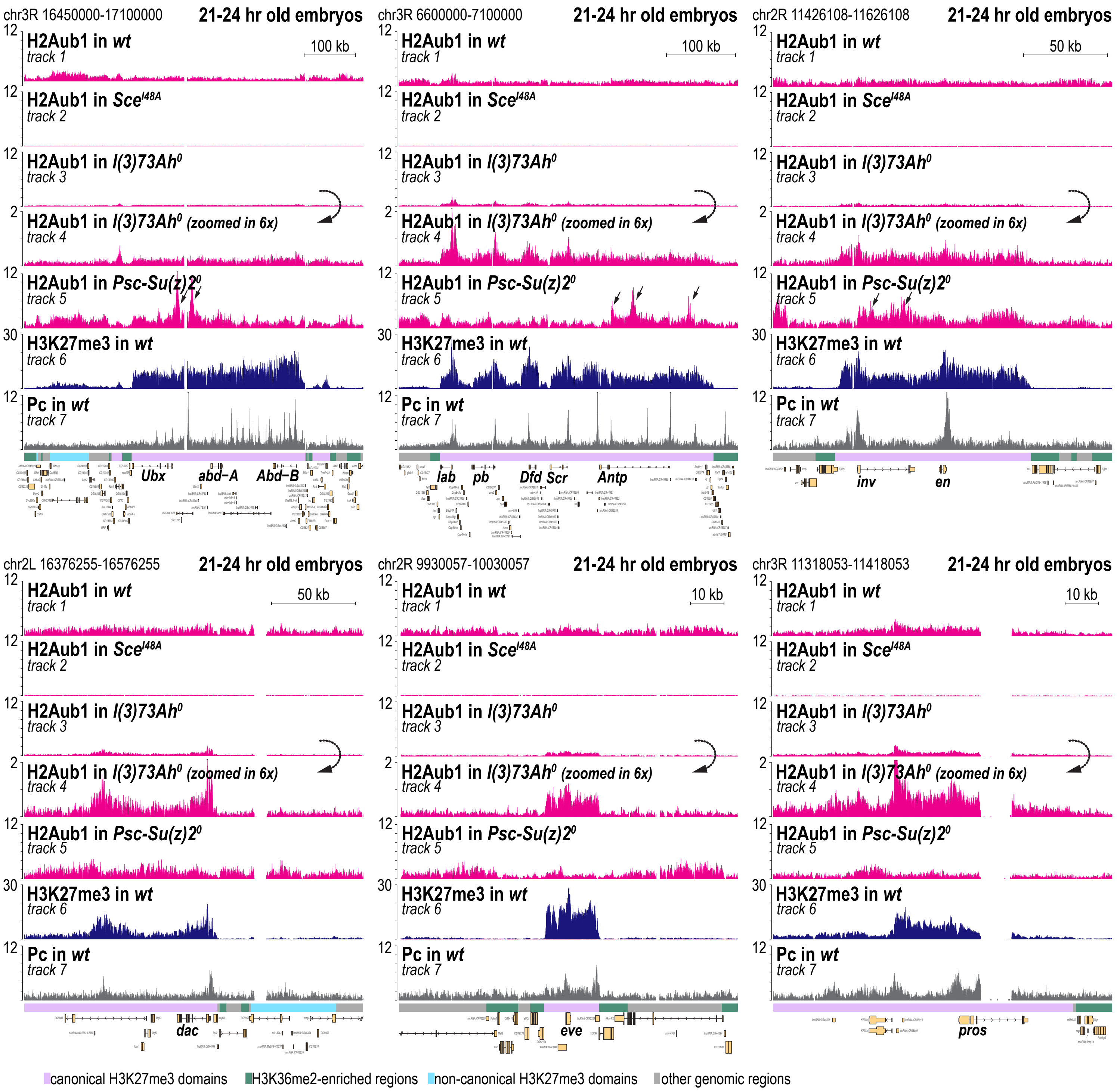

chr3R 16450000-17100000
21-24 hr old embryos
100 kb
H2Aub1 in wt
track 1
H2Aub1 in Sce^I48A
track 2
H2Aub1 in l(3)73Ah^0
track 3
H2Aub1 in l(3)73Ah^0 (zoomed in 6x)
track 4
H2Aub1 in Psc-Su(z)2^0
track 5
H3K27me3 in wt
track 6
Pc in wt
track 7
Ubx
abd-A
Abd-B
chr3R 6600000-7100000
21-24 hr old embryos
100 kb
lab
pb
Dfd
Scr
Antp
chr2R 11426108-11626108
21-24 hr old embryos
50 kb
inv
en
chr2L 16376255-16576255
21-24 hr old embryos
50 kb
dac
chr2R 9930057-10030057
21-24 hr old embryos
10 kb
eve
chr3R 11318053-11418053
21-24 hr old embryos
10 kb
pros
canonical H3K27me3 domains
H3K36me2-enriched regions
non-canonical H3K27me3 domains
other genomic regions

Figure S2

H2Aub1 profiles at selected Polycomb target genes in *Sce^{I48A}*, *l(3)73Ah^{0}* and *Psc-Su(z)2^{0}* embryos

H2Aub1 (tracks 1-5), H3K27me3 (track 6) and Pc (track 7) ChIP-seq profiles in 21-24 hr old embryos of the indicated genotypes as in Fig. 1B but at chromosomal intervals containing the Polycomb target genes *Ubx, abd-A and Abd-B* in the *Bithorax-Complex, lab, pb, Dfd, Scr* and *Antp* in the *Antennapedia-Complex*, *inv* and *en, dac, eve, pros*. Arrows in track 5 mark regions with high *de novo* enrichment of H2Aub1; the molecular basis for this accumulation is not known by note that these regions do not correspond to PRE sites bound by Pc (compare with track 7).

Figure S3

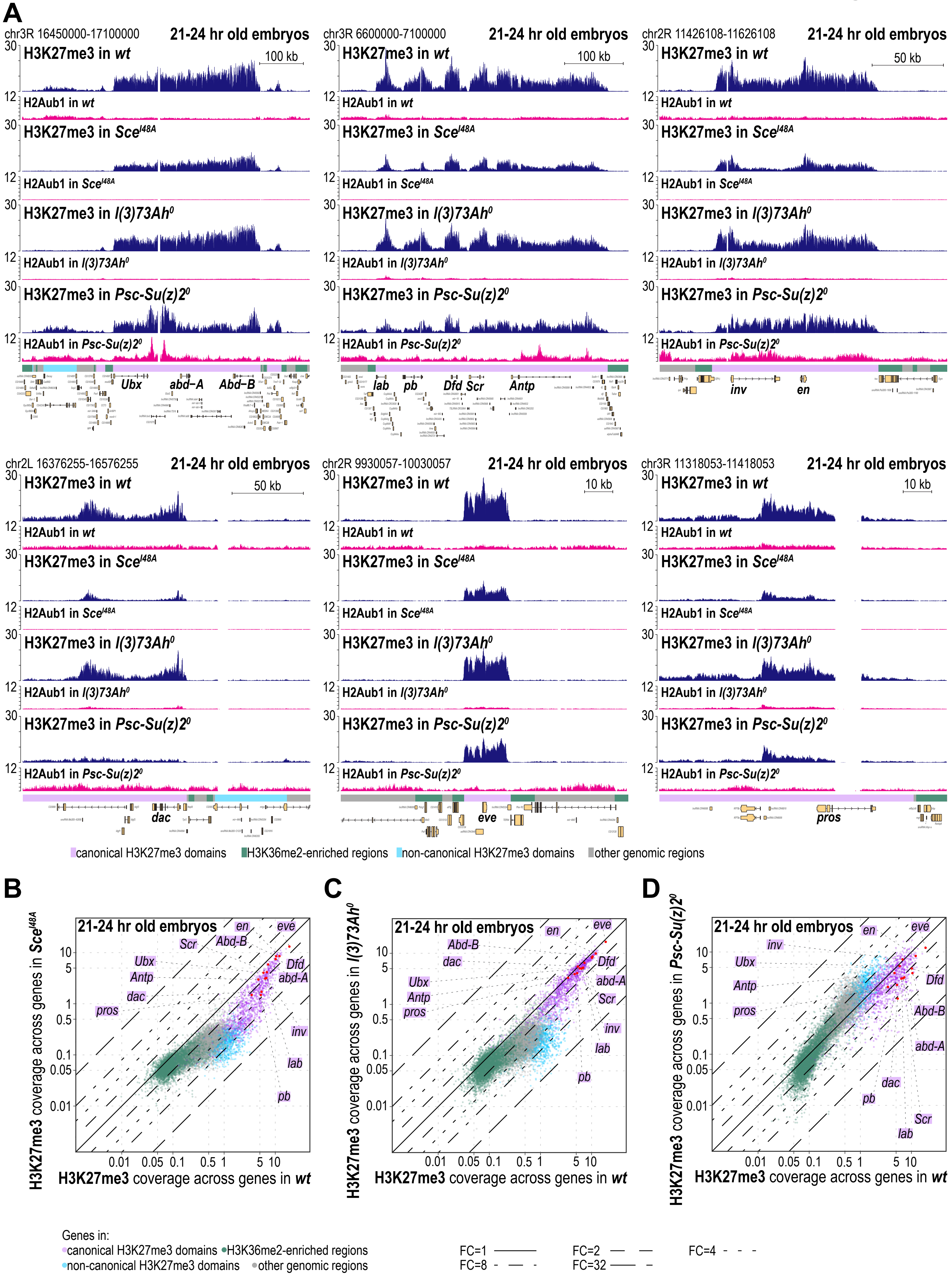

A
chr3R 16450000-17100000
21-24 hr old embryos
H3K27me3 in wt
100 kb
H2Aub1 in wt
H3K27me3 in Sce^I48A
H2Aub1 in Sce^I48A
H3K27me3 in l(3)73Ah^0
H2Aub1 in l(3)73Ah^0
H3K27me3 in Psc-Su(z)2^0
H2Aub1 in Psc-Su(z)2^0
Ubx
abd-A
Abd-B
chr3R 6600000-7100000
lab
pb
Dfd
Scr
Antp
chr2R 11426108-11626108
50 kb
inv
en
chr2L 16376255-16576255
dac
chr2R 9930057-10030057
10 kb
eve
chr3R 11318053-11418053
pros
canonical H3K27me3 domains
H3K36me2-enriched regions
non-canonical H3K27me3 domains
other genomic regions
B
C
D
H3K27me3 coverage across genes in wt
H3K27me3 coverage across genes in Sce^I48A
H3K27me3 coverage across genes in l(3)73Ah^0
H3K27me3 coverage across genes in Psc-Su(z)2^0
Genes in:
canonical H3K27me3 domains
H3K36me2-enriched regions
non-canonical H3K27me3 domains
other genomic regions
FC=1
FC=2
FC=4
FC=8
FC=32

Figure S3

H3K27me3 profiles in *Sce*^{I48A}, *l(3)73Ah*^{0} and *Psc-Su(z)2*^{0} mutant embryos

(A) H3K27me3 and H2Aub1 ChIP-seq profiles in 21-24 hr old embryos of the indicated genotypes at the same chromosomal intervals as in Fig. S2, containing the indicated Polycomb target genes *Ubx*, *abd-A* and *Abd-B* in the *Bithorax-Complex*, *lab*, *pb*, *Dfd*, *Scr* and *Antp* in the *Antennapedia-Complex*, *inv* and *en*, *dac*, *eve*, *pros*.

In *Sce*^{I48A} mutants, H3K27me3 levels were markedly reduced at most of these target genes but *Antp*, *Ubx*, *Abd-B* and *en* nevertheless remained fully repressed outside of their normal expression domains (Pengelly et al, 2015; Fig. 2). We note that in *Sce*^{0} (*Sce*$^{mat- zyg-}$) mutants, expression of the *pros*, *eve* and *dac* genes also remained confined to their normal expression domains (Gutierrez et al, 2012).

In *l(3)73Ah*^{0} mutants, H3K27me3 levels were at most regions only very mildly reduced compared to wildtype.

In *Psc-Su(z)*^{0} mutants, H3K27me3 levels were markedly or even drastically reduced at the regions shown here, whereas H2Aub1 levels were not diminished (see main text and Fig. S2). Note that *Scr*, *Antp*, *Ubx*, *abd-A*, *Abd-B*, *pros*, *eve*, *en* and *dac* are all widely misexpressed in *Psc-Su(z)*^{0} mutant embryos (Soto et al, 1995; Gutierrez et al, 2012; Fig. 2). The reduction in H3K27me3 levels at these genes may thus be a consequence of the widespread upregulation of expression of these genes in many embryonic cells or another secondary effect due to the extensive cell fate changes and developmental abnormalities (Fig. 2) in these embryos.

(B) Scatter plot showing H3K27me3 read coverage across gene bodies in 21-24 hr old wild-type (*wt*) and *Sce*^{I48A} mutant embryos, as in Fig. 3B.

(C) Scatter plot as in (B) but comparing wild-type and *l(3)73Ah*^{0} mutant embryos.

(D) Scatter plot as in (B) but comparing wild-type and *Psc-Su(z)*^{0} mutant embryos.

Figure S4

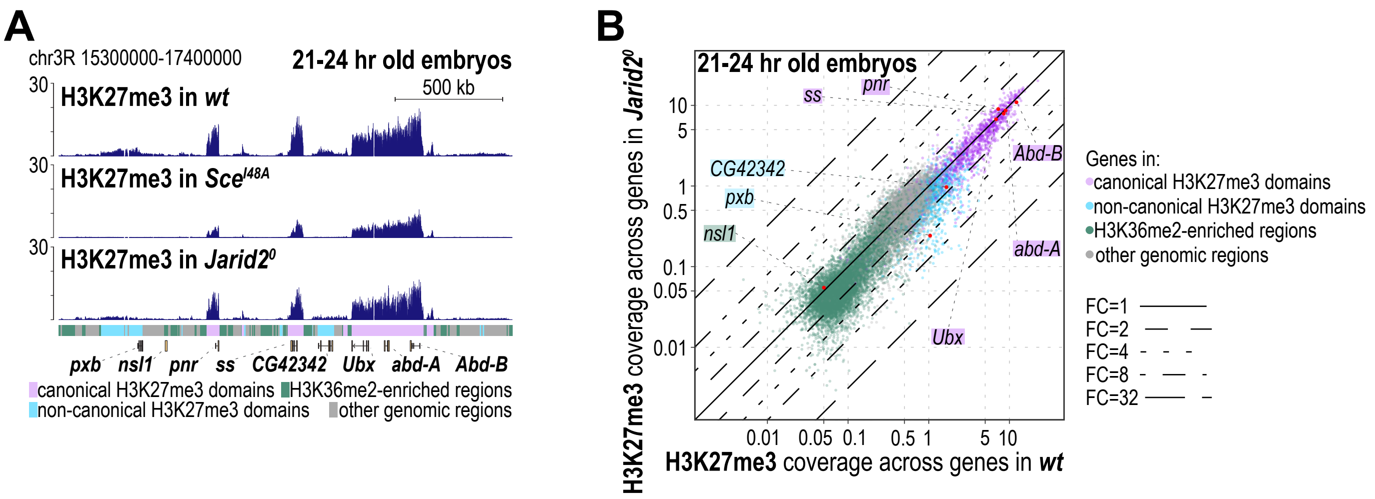
A
chr3R 15300000-17400000
21-24 hr old embryos
500 kb
30
H3K27me3 in wt
30
H3K27me3 in Sce[I48A]
30
H3K27me3 in Jarid2[0]
pxb nsl1 pnr ss CG42342 Ubx abd-A Abd-B
canonical H3K27me3 domains
H3K36me2-enriched regions
non-canonical H3K27me3 domains
other genomic regions
B
21-24 hr old embryos
ss
pnr
CG42342
pxb
nsl1
Abd-B
abd-A
Ubx
10
5
1
0.5
0.1
0.05
0.01
0.01 0.05 0.1 0.5 1 5 10
H3K27me3 coverage across genes in wt
H3K27me3 coverage across genes in Jarid2[0]
Genes in:
canonical H3K27me3 domains
non-canonical H3K27me3 domains
H3K36me2-enriched regions
other genomic regions
FC=1
FC=2
FC=4
FC=8
FC=32

Figure S4

H3K27me3 profiles in *Jarid2^{0}* mutant embryos

(A) H3K27me3 ChIP-seq profiles in 21-24 hr old embryos of the indicated genotypes at the same chromosomal interval as in Fig. 1B.

(B) Scatter plot showing H3K27me3 read coverage across gene bodies in 21-24 hr old wild-type (*wt*) and *Jarid2^{0}* mutant embryos.

Jarid2^{0} mutants showed no major reduction of H3K27me3 levels at canonical H3K27me3 domains and only a subset of non-canonical H3K27me3 domains showed a greater than two-fold reduction in H3K27me3 coverage; compare with H3K27me3 track in *Sce^{I48A}* mutants in (A), for scatter plot comparing *Sce^{I48A}* mutants and wildtype see Fig. 3B. Note that in *Jarid2^{0}* mutants, PRC2.2 is still expected to retain AEBP2 (Jing) protein and thus also H2Aub1 binding.
