# Supplementary material for: Histone modification cross-talk and protein complex diversification confer plasticity to Polycomb repression
Source: Genes Dev. 2026 Jan 1;40(1-2):43–55. doi: 10.1101/gad.353148.125 (PMC12758141; doi:10.1101/gad.353148.125)
Supplement: Supplement 2 [file Supplemental_TableS1.pdf]

Table S1

Mutant alleles and genotypes of animals used in the different figures

The following **mutant alleles** were used in this study:

Sce^{KO}: null allele (Gutiérrez et al, 2012), referred to as *Sce*^{0} in this manuscript.

l(3)73Ah^{0}: generated in this study, see Material and Methods for details.

Psc-Su(z)2^{0}: generated in this study, see Material and Methods for details.

esc^{2}: null allele (Struhl, 1981), referred to as *esc*^{0} in this manuscript.

esc^{6}: null allele (Struhl, 1981), referred to as *esc*^{0} in this manuscript.

Pcl^{22M21}: null allele (Nekrasov et al, 2007), referred to as *Pcl*^{0} in this manuscript.

E(z)^{731}: null allele (Müller et al, 2002), referred to as *E(z)*^{0} in this manuscript.

Asx^{22P4}: null allele (Scheuermann et al, 2010) referred to as *Asx*^{0} in this manuscript.

Asx^{27J6}: null allele (Scheuermann et al, 2010) referred to as *Asx*^{0} in this manuscript.

Jarid2^{KO}: null allele (Shalaby et al, 2017), referred to as *Jarid2*^{0} in this manuscript.

Genotypes of animals used in the different figures

Figure 1, S1 and 2:

wt: Oregon-R

Sce^{I48A}: obtained as GFP-negative progeny in crosses from mothers and fathers of the following genotype:

w; Sce^{I48A} *(VK37) >Sce*$^{+}$*> (J27) / Sce*^{I48A}*(VK37); nos-Gal4VP16 F82B cu sr Sce*^{0} */ UAS-FLP(VK33) F82B cu sr Sce*^{0} (see (Pengelly et al, 2015) for more details)

l(3)73Ah^{0}: obtained as GFP-negative progeny from heat-shocked mothers that were:

yw hs-Flp122 / w; +; ovo^{D1} *w+ F2A / l(3)73Ah*^{0} *F2A* and were crossed to *w / Y; +; l(3)73Ah*^{0} *F2A / TM3 twi-Gal4 UAS-GFP* fathers.

Psc-Su(z)2$^{\mathbf{0}}$*:* obtained as GFP negative progeny from mothers and fathers of the following genotype:

w; F42D y+ Psc-Su(z)2^{0} */ Cyo twi-Gal4 UAS-GFP*

Figure 3A-E:

wt: Oregon-R

esc$^{\mathbf{0}}$: obtained as progeny in crosses from mothers and fathers of the following genotype:

yw / + or Y; esc^{6} *b pr / CyO, esc*^{2}.

Sce$^{\mathbf{I48A}}$: obtained as GFP-negative progeny in crosses from mothers and fathers of the following genotype:

w; Sce^{I48A} *(VK37) >Sce*$^{+}$*> (J27) / Sce*^{I48A}*(VK37); nos-Gal4VP16 F82B cu sr Sce*^{0} */ UAS-FLP(VK33) F82B cu sr Sce*^{0} (see (Pengelly et al, 2015) for more details)

Pcl$^{\mathbf{0}}$*:* obtained as GFP negative progeny from mothers and fathers of the following genotype:

w; F40 F42D y^{+} *Pcl*^{0} */ Cyo twi-Gal4 UAS-GFP*

Figure 3F-G:

wt: imaginal discs from 3rd instar larvae of the following genotype:

yw hs-flp122; y^{+} *hs-nGFP FRT2A*

Sce$^{\mathbf{I48A\ z-}}$: imaginal discs from 3rd instar larvae of the following genotype:

w; Sce^{I48A}*; F82B Sce*^{0}

Figure 3H:

Pcl$^{\mathbf{0}}$ ***clones in Sce***$^{\mathbf{I48A\ z-}}$: GFP-negative clones of *Pcl*^{0} homozygous cells were induced by Flp expression in animals of the following genotype:

yw hs-Flp122; Sce^{I48A} *(VK37) F42D Pcl*^{0} */ F42D hs-nGFP; F82B Sce*^{0}*/ F82B Sce*^{0}

Figure 3I:

Pcl^0: GFP-negative clones of *Pcl^0* homozygous cells were induced by Flp expression in animals of the following genotype:

yw hs-Flp122; F40 F42D y^+Pcl^0 / F42D hs-nGFP

Figure 3J:

$E(z)^0$: GFP-negative clones of *$E(z)^0$* homozygous cells were induced by Flp expression in animals of the following genotype:

yw hs-Flp122; +; $E(z)^0$ F2A / hs-nGFP F2A

Figure 4:

wt: Oregon-R

Asx^0: obtained as GFP negative progeny from mothers and fathers of the following genotype:

w; F40 F42D y^+ Asx^0 / Cyo twi-Gal4 UAS-GFP

Asx^0 Pcl^0: obtained as GFP negative progeny from mothers and fathers of the following genotype:

w; F40 F42D y^+ Asx^0 Pcl^0 / Cyo twi-Gal4 UAS-GFP

Pcl^0: obtained as GFP negative progeny from mothers and fathers of the following genotype:

w; F40 F42D y^+ Pcl^0 / Cyo twi-Gal4 UAS-GFP

Figure 5:

wt: Oregon-R

Pcl^0: obtained as GFP negative progeny from mothers and fathers of the following genotype:

w; F40 F42D y^+ Pcl^0 / Cyo twi-Gal4 UAS-GFP

***Asx*^{0}**: obtained as GFP negative progeny from mothers and fathers of the following genotype:

w; F40 F42D y^{+} *Asx*^{0} */ Cyo twi-Gal4 UAS-GFP*

***Asx*^{0} *Pcl*^{0}**: obtained as GFP negative progeny from mothers and fathers of the following genotype:

w; F40 F42D y^{+} *Asx*^{0} *Pcl*^{0} */ Cyo twi-Gal4 UAS-GFP*

***Asx*^{0} *Pcl*^{0} *Jarid2*^{0}**: obtained as GFP-negative progeny from heat-shocked mothers that were: *yw hs-Flp122 / w; F40 F42D y*$^{+}$ *Asx*^{0} *Pcl*^{0} */ Cyo twi-Gal4 UAS-GFP; ovo*^{D1} *w*$^{+}$ *F2A / Jarid2*^{0} *F2A* and were crossed to *F40 F42D y*$^{+}$ *Asx*^{0} *Pcl*^{0} */ Cyo twi-Gal4 UAS-GFP; Jarid2*^{0} *F2A / TM3 twi-Gal4 UAS-GFP*

***Jarid2*^{0}**: obtained as GFP-negative progeny from heat-shocked mothers that were: *yw hs-Flp122 / w; +; ovo*^{D1} *w+ F2A / Jarid2*^{0} *F2A* and were crossed to *w / Y; +; Jarid2*^{0} *F2A / TM3 twi-Gal4 UAS-GFP* fathers.

***Asx*^{0} *Jarid2*^{0}**: obtained as GFP-negative progeny from heat-shocked mothers that were: *yw hs-Flp122 / w; F40 F42D y*$^{+}$ *Asx*^{0} */ Cyo twi-Gal4 UAS-GFP; ovo*^{D1} *w*$^{+}$ *F2A / Jarid2*^{0} *F2A* and were crossed to *F40 F42D y*$^{+}$ *Asx*^{0} */ Cyo twi-Gal4 UAS-GFP; Jarid2*^{0} *F2A / TM3 twi-Gal4 UAS-GFP*
