# Supplementary material for: Histone modification cross-talk and protein complex diversification confer plasticity to Polycomb repression
Source: Genes Dev. 2026 Jan 1;40(1-2):43–55. doi: 10.1101/gad.353148.125 (PMC12758141; doi:10.1101/gad.353148.125)
Supplement: Supplement 3 [file Supplemental_TableS2.pdf]

Table S2

Antibodies used in this study

Specificity	Source / Reference
H2AK119ub	Cell Signaling (D27C4)
H3K27me3	Cell Signaling (C36B11)
Pc	Papp and Müller 2006
Abd-B	DSHB (1A2E9)
Antp	DSHB (8C11)
Ubx	DSHB (FP3.38)
GFP	Aves Labs (GFP-1020)
